# Axillary lymph node status and invasive lobular breast cancer: Analysis of the Clinical Tumor Register of the AGO Austria

**DOI:** 10.1007/s00508-023-02162-y

**Published:** 2023-04-03

**Authors:** Sabine Danzinger, Karin Pöckl, Gerit Kronawetter, Christian Pfeifer, Sandra Behrendt, Patricia Gscheidlinger, Lois Harrasser, Helmut Mühlböck, Walter Dirschlmayer, Christian Schauer, Roland Reitsamer, Heidemarie Uher, Kristina Schönau, Irmgard Delmarko, Christian F. Singer

**Affiliations:** 1 Department of Obstetrics and Gynecology, Medical University of Vienna, Spitalgasse 23, 1090 Vienna, Austria; 2 Department of Statistics, University of Innsbruck, Innsbruck, Austria; 3 Department of Clinical Epidemiology, Tyrolean Federal Institute for Integrated Care, Tirol Kliniken GmbH, Innsbruck, Austria; 4 Department of Obstetrics and Gynecology, Hospital Barmherzige Schwestern Ried, Ried im Innkreis, Austria; 5 Department of Gynecology, Hospital Barmherzige Brüder Graz, Graz, Austria; 6 Department of Gynecology, Paracelsus Medical University, University Hospital Salzburg, Landeskrankenhaus Salzburg, Salzburg, Austria; 7 Department of Surgery, Breast Health Center, Hospital Landstraße, Vienna, Austria; 8 Department of General, Visceral and Tumor Surgery, Breast Health Center, Hospital Ottakring, Vienna, Austria

**Keywords:** Invasive breast cancer, Invasive lobular carcinoma, Invasive ductal carcinoma, Pathological node stage, Axillary lymph node metastasis

## Abstract

**Background:**

Invasive lobular carcinoma (ILC) represents the second most common type of invasive breast cancer (BC). Although ILC generally have good prognostic properties (positive estrogen receptor, ER, low tumor grade), they are generally diagnosed at a more advanced stage. The data on the axillary lymph node status in ILC compared to invasive ductal carcinoma (IDC) are considered controversial. Therefore, the aim of this study was to compare the pathological node stage (pN) between ILC and IDC in an Austria-wide register.

**Methods:**

Data of the Clinical Tumor Register (Klinisches TumorRegister, KTR) of the Austrian Association for Gynecological Oncology (AGO) were retrospectively analyzed. Patients with primary early BC, invasive lobular or ductal, diagnosed between January 2014 and December 2018, and primary surgery were included. A total of 2127 tumors were evaluated and compared in 2 groups, ILC *n* = 303, IDC *n* = 1824.

**Results:**

A total of 2095 patients were analyzed in the study. In the multivariate analysis, pN2 and pN3 were observed significantly more frequently in ILC compared with IDC (odds ratio, OR 1.93; 95% confidence interval, CI 1.19–3.14; *p* = 0.008 and OR 3.22; 95% CI: 1.47–7.03; *p* = 0.003; respectively). Other factors associated with ILC were tumor grades 2 and 3, positive ER, and pathological tumor stage (pT) 2 and pT3. In contrast, concomitant ductal carcinoma in situ, overexpression of the human epidermal growth factor receptor 2 (HER2), and a moderate and high proliferation rate (Ki67) were found less frequently in ILC.

**Conclusion:**

The data show an increased risk of extensive axillary lymph node metastasis (pN2/3) in ILC.

## Background

Invasive breast cancer (BC) is considered a heterogeneous disease. After invasive ductal carcinoma (IDC), invasive lobular carcinoma (ILC) represents the second most common type of invasive BC, comprising up to 15% of all cases [1–3].

The ILC is characterized by diffuse infiltration of the stroma with a single file pattern. The loss or lack of epithelial E‑cadherin expression represents a characteristic feature of ILC. E‑cadherin mediates cell-cell adhesion and dysregulation results in distinctive discohesive growth patterns observed in ILC. Thus, E‑cadherin antibodies are used to differentiate between lobular and ductal lesions by immunohistochemistry [4, 5].

The ILC distinctly differs from IDC in its clinicopathological characteristics and molecular alterations. Compared to IDC, ILC is more frequently multifocal, multicentric, and bilateral. Many studies show that ILC is associated with a good prognostic phenotype such as positive hormone receptors (HR) (both estrogen and progesterone receptors, ER, PR), human epidermal growth factor receptor 2 (HER2)-negativity, low tumor grade, and a low to moderate proliferation index. Despite these good prognostic properties, invasive lobular tumors are generally diagnosed at a more advanced stage. Furthermore, there is a tendency for late recurrences, and a higher rate of multiple metastases with a variable pattern of involvement of distant sites [3, 6–18].

The axillary lymph node (ALN) involvement represents one of the most important prognostic factors in early BC [19–25]; however, previous studies have shown controversial results regarding the ALN status between ILC and IDC. There are many studies demonstrating that the ALN involvement does not differ between both groups [10, 26, 27]; however, some studies showed an association between ILC and a higher incidence of positive ALN involvement [8, 28, 29]. In contrast, there are studies reporting less frequent ALN positivity in ILC [30, 31]. Therefore, the aim of this study was to compare the pathological node stage (pN) between ILC and IDC in an Austria-wide register.

## Methods

### Study population

This was a retrospective study based on data from patients of the Clinical Tumor Register (Klinisches TumorRegister, KTR) of the Austrian Association for Gynecological Oncology (AGO). Women with primary early BC, invasive lobular or ductal, diagnosed between January 2014 and December 2018, and primary surgery were enrolled in the study. Exclusion criteria were patients with both ductal and lobular carcinoma, patients who had neoadjuvant therapy, patients with recurrent disease, and patients with primary metastatic disease. In cases of bilateral BC, both sides were included as separate tumors. Altogether, the analysis was based on 2095 patients (bilateral cancer *n* = 32), all 2127 tumors were evaluated and compared in 2 groups, ILC *n* = 303 (14.2%), IDC *n* = 1824 (85.8%).

The KTR represents a computerized database and cancer register collecting data on breast, endometrial, ovarian, and cervical cancer from hospitals throughout Austria (https://ktr.iet.at). This register is maintained by the Department of Clinical Epidemiology, Tyrolean Federal Institute of Integrated Care, Innsbruck, Austria (https://www.iet.at).

In this study, the following clinicopathological parameters of BC patients were evaluated: age at diagnosis, menopausal status, bilateral cancer, tumor morphology, concomitant ductal carcinoma in situ (DCIS) component, tumor grade, immunohistochemical parameters (ER, PR, HER2, Ki67, ≤ 14% low, 15–40% intermediate, > 40% high), lymphovascular invasion (LVI), blood vessel invasion (BVI), and pathological tumor (pT) stage and pathological node (pN) stage. Additionally, we evaluated the axillary dissection rate, and the total number of lymph nodes removed. Comparisons were then made between patients/tumors divided into two separate histologic subgroups: IDC and ILC.

### Statistical analysis

Descriptive statistics were performed to analyze the characteristics of the two histopathological groups (ILC and IDC). The χ^2^ and Fisher’s exact (for smaller sample sizes) tests were used to investigate the proportions of the clinical histopathological characteristics between ILC and IDC. Logistic regression was performed to identify independent parameters associated with ILC and IDC. Associations were summarized using the odds ratios (OR) and corresponding 95% CI derived from the model estimates. We excluded all unknown or undetermined values from the analysis. Statistical significance was considered at *p* < 0.05 (two-tailed). We performed all statistical analyses using the statistical software package R version 3.4.1 [32].

### Ethics approval

This study was approved by the ethics committee of the Medical University of Vienna (1589/2019). All procedures performed in the study were in accordance with the ethical standards of the institutional committee and with the 1964 Declaration of Helsinki and its later amendments or comparable ethical standards. According to the ethics committee of the Medical University of Vienna, written informed consent was not required owing to the retrospective design of the study.

## Results

Altogether 2095 patients were evaluated in the study. The study population comprised 2 subgroups: 297 patients with ILC (14.2%) and 1798 patients with IDC (85.8%). The characteristics of the patients are summarized in Table 1. The mean age at diagnosis was 65.0 years (standard deviation (SD): 12.4) in patients with ILC compared to 63.0 years (SD: 12.9) in IDC patients (*p* = 0.01). We found no significant difference in the distributions of age at diagnosis between both groups (*p* = 0.27). Premenopausal patients were significantly more common in the IDC (*n* = 402, 22.4%) compared to the ILC group (*n* = 49, 16.5%) (*p* = 0.03). Bilateral BC was diagnosed in 32 (1.5%) patients; bilateral cancers did not differ between the groups (*p* = 0.62).

**Table 1:** Characteristics of the patients and tumors

**Patients**_**total**_** (*n*** **=** **2095)**						
**–**	**ILC (*n*** **=** **297)**		**IDC (*n*** **=** **1798)**			***p‑value*** ^a^
Age at diagnosis (years)						
Mean (SD)	65.0 (12.4)		63.0 (12.9)			*0.01*
*–*	*n*	*%*	*n*		*%*	*–*
20–39	4	1.3	44		2.4	0.27
40–59	100	33.7	684		38.0	–
60–79	159	53.5	888		49.4	–
80–99	34	11.4	182		10.1	–
Menopausal status						
Premenopausal	49	16.5	402		22.4	*0.03*
Postmenopausal	229	77.1	1289		71.7	–
Unknown	19	6.4	107		6.0	–
Bilateral BC						
Yes	6	2.0	26		1.4	0.62
No	291	98.0	1772		98.6	–
**Tumors**_**total**_** (*n*** **=** **2127)**						
–	**ILC (*n*** **=** **303)**		**IDC (*n*** **=** **1824)**			–
*–*	*n*	*%*	*n*		*%*	***p‑value*** ^*a*^
DCIS component						
Yes	67	22.1	915		50.2	*<* *0.001*
No	236	77.9	905		49.6	–
Unknown	0	0.0	4		0.2	–
Tumor grade						
1	29	9.6	399		21.9	*<* *0.001*
2	218	71.9	995		54.6	–
3	51	16.8	418		22.9	–
Unknown	5	1.7	12		0.7	–
ER						
Positive	298	98.3	1529		83.8	*0.001*
Negative	4	1.3	100		5.5	–
Unknown	1	0.3	195		10.7	–
PR						
Positive	255	84.2	1399		76.7	0.50
Negative	47	15.5	226		12.4	–
Unknown	1	0.3	199		10.9	–
HER2						
Positive	15	5.0	228		12.5	*<* *0.001*
Negative	282	93.1	1576		86.4	–
Unknown	6	2.0	20		1.1	–
Ki67						
Low	158	52.1	670		36.7	*<* *0.001*
Moderate	122	40.3	684		37.5	–
High	18	5.9	244		13.4	–
Unknown	5	1.7	226		12.4	–
LVI						
Yes	56	18.5	348		19.1	0.97
No	230	75.9	1402		76.9	–
Unknown	17	5.6	74		4.1	–
BVI						
Yes	6	2.0	33		1.8	0.88
No	247	81.5	1622		88.9	–
Unknown	50	16.5	169		9.3	–
pT stage						
pT0	0	0.0	0		0.0	*<* *0.001*
pTis	0	0.0	0		0.0	–
pT1	170	56.1	1332		73.0	–
pT1mic	1	0.3	13		0.7	–
pT1a	10	3.3	97		5.3	–
pT1b	39	12.9	393		21.5	–
pT1c	120	39.6	829		45.4	–
pT2	98	32.3	436		23.9	–
pT3	29	9.6	35		1.9	–
pT4	5	1.7	18		1.0	–
pT4a	0	0.0	2		0.1	–
pT4b	5	1.7	16		0.9	–
pT4c	0	0.0	0		0.0	–
pT4d	0	0.0	0		0.0	–
pTX	1	0.3	3		0.2	–
pN stage						
pN0	200	66.0	1235		67.7	*<* *0.001*
pN0	198	65.3	1224		67.1	–
pN0 (i+)	2	0.7	11		0.6	–
pN1	54	17.8	429		23.5	–
pN2	24	7.9	75		4.1	–
pN2a	23	7.6	74		4.1	–
pN2b	1	0.3	1		0.1	–
pN3	10	3.3	19		1.0	–
pN3a	10	3.3	19		1.0	–
pN3b	0	0.0	0		0.0	–
pN3c	0	0.0	0		0.0	–
***pN1‑3***	88	29.0	523		28.7	0.84
pNX	15	5.0	66		3.6	–

*BC* breast cancer, *BVI* blood vessel invasion, *DCIS* ductal carcinoma in situ, *ER* estrogen receptor, *HER2* human epidermal growth factor receptor 2, *IDC* invasive ductal carcinoma, *ILC* invasive lobular carcinoma, *Ki67* marker of proliferation Ki67, *LVI* lymphovascular invasion, *pN* pathological node stage, *pNX* unknown pathological node stage, *PR* progesterone receptor, *pT* pathological tumor stage, *pTX* unknown pathological tumor stage, *SD* standard deviation

^a^ ILC versus IDC, χ^2^or Fisher’s exact test; unknown/undetermined values were excluded from the analysis

With respect to the tumors included, a concomitant DCIS component was found in 67 (22.1%) of ILC versus 915 (50.2%) of IDC (*p* < 0.001). Grade 2 was the most common tumor grade in both groups (218, 71.9% of ILC, 995, 54.6% of IDC, *p* < 0.001). Positive expression of ER was significantly more common among lobular tumors (*n* = 298, 98.3%) compared to IDC (*n* = 1529, 83.8%, *p* = 0.001). With respect to PR, there was no significant difference observed among the groups and HER2 was positive in only 15 (5.0%) of ILC versus 228 (12.5%) of invasive ductal tumors (*p* < 0.001). The proliferation rate (Ki67) was significantly different between both subgroups, we found a higher proportion of low and moderate Ki67 in ILC (*n* = 158, 52.1% and *n* = 122, 40.3%, respectively) in comparison with IDC (*n* = 670, 36.7% and *n* = 684, 37.5%, respectively) (*p* < 0.001). Regarding lymphovascular and blood vessel invasion (LVI, BVI), there was no significant difference between the histological subgroups.

In contrast, the pT and pN distribution differed significantly between ILC and IDC (both *p* < 0.001). The pT1 stage was found in 170 (56.1%) of lobular cancers versus 1332 (73.0%) of IDC. Invasive lobular tumors were more often diagnosed at a higher pT stage (pT2‑4, *n* = 132, 43.6%) compared to invasive ductal cases (*n* = 489, 26.8%). The most common pN stage was pN0 in both groups (200, 66.0% of ILC, 1235, 67.7% of IDC). The proportion of a positive pN stage (pN1-3) did not differ significantly between invasive lobular and ductal tumors (88, 29.0% versus 523, 28.7%, respectively, *p* = 0.84).

An axillary lymph node dissection was performed in 94 (31.0%) of invasive lobular versus 509 (27.9%) of invasive ductal carcinomas (*p* = 0.18). The mean total number of lymph nodes removed was 5.2 (SD: 5.6) in lobular versus 4.8 (SD: 5.1) in ductal tumors (*p* = 0.79) (Table 2).

**Table 2:** Axillary management: axillary lymph node dissection and total number of lymph nodes removed

	Tumors_total_ (*n* = 2127)				
	ILC (*n* = 303)		IDC (*n* = 1824)		
	n	%	n	%	*p‑value* ^a^
**Axillary lymph node dissection**					
Yes	94	31.0	509	27.9	0.18
No	201	66.3	1306	71.6	–
Unknown	8	2.6	9	0.5	–
**Total number of lymph nodes removed**					
Evaluable tumors	275	90.8	1723	94.5	–
Total number of lymph nodes removed	1427	–	8296	–	–
Mean (SD)	5.2 (5.6)	–	4.8 (5.1)	–	0.79

*IDC* invasive ductal carcinoma, *ILC* invasive lobular carcinoma, *SD* standard deviation

^a^ ILC versus IDC, χ^2^ or Fisher’s exact test; unknown/undetermined values were excluded from the analysis

In a univariate analysis of clinical pathological factors associated with ILC versus IDC, age at diagnosis, menopausal status, concomitant DCIS component, tumor grade, ER, HER2, Ki67, and pT and pN stages were found to be independent parameters; however, when these features were put into a multivariate regression model and adjusted for age, the following parameters were observed significantly more frequently in ILC compared with IDC: pN2 and pN3 (OR: 1.93; 95% CI: 1.19–3.14; *p* = 0.008; OR: 3.22; 95% CI: 1.47–7.03; *p* = 0.003, respectively), tumor grades 2 and 3 (OR: 2.99; 95% CI: 1.99–4.48; *p* < 0.001; OR: 1.63; 95% CI: 1.01–2.62; *p* = 0.045, respectively), positive ER (OR: 5.07; 95% CI: 1.85–13.88; *p* = 0.002), and pT2 and pT3 (OR: 1.72; 95% CI: 1.31–2.26; *p* < 0.001; OR: 6.31; 95% CI: 3.75–10.60; *p* < 0.001, respectively). In contrast, concomitant DCIS (OR: 0.29; 95% CI: 0.21–0.38; *p* < 0.001), HER2-positivity (OR: 0.36; 95% CI: 0.21–0.61; *p* < 0.001), and moderate or high Ki67 (OR: 0.76; 95% CI: 0.58–0.98; *p* = 0.034; OR: 0.31; 95% CI: 0.19–0.52; *p* < 0.001, respectively) were found less frequently in ILC (Table 3).

**Table 3:** Logistic regression analysis of ILC versus IDC: univariate and multivariate analysis

Characteristic		Univariate LR	*p*-value	Multivariate LR ^a^	*p*-value
		OR (95% CI)		OR (95% CI)	
	ILC vs. IDC				
Age at diagnosis					
(years)	–	1.01 per year (1.00–1.02)	*0.011*	–	–
–	20–39	1.0	–	–	–
–	40–59	1.61 (0.57–4.57)	0.373	–	–
–	60–79	1.97 (0.70–5.56)	0.200	–	–
–	80–99	2.05 (0.69–6.09)	0.194	–	–
Menopausal status	Premenopausal	1.0	–	–	–
	Postmenopausal	1.46 (1.05–2.02)	*0.024*	1.11 (0.70–1.76)	0.654
Bilateral BC	No	1.0	–	–	–
	Yes	1.41 (0.57–3.44)	0.457	1.32 (0.54–3.24)	0.550
DCIS component	No	1.0	–	–	–
	Yes	0.28 (0.21–0.37)	*<* *0.001*	0.29 (0.21–0.38)	*<* *0.001*
Tumor grade	1	1.0	*–*	–	–
	2	3.01 (2.01–4.52)	*<* *0.001*	2.99 (1.99–4.48)	*<* *0.001*
	3	1.68 (1.04–2.70)	*0.033*	1.63 (1.01–2.62)	*0.045*
ER	Negative	1.0	–	–	–
	Positive	4.87 (1.78–13.33)	*0.002*	5.07 (1.85–13.88)	*0.002*
PR	Negative	1.0	–	–	–
	Positive	0.88 (0.62–1.23)	0.449	0.92 (0.65–1.29)	0.616
HER2	Negative	1.0	–	–	–
	Positive	0.37 (0.21–0.63)	*<* *0.001*	0.36 (0.21–0.61)	*<* *0.001*
Ki67	Low	1.0	–	–	–
	Moderate	0.76 (0.58–0.98)	*0.035*	0.76 (0.58–0.98)	*0.034*
	High	0.31 (0.19–0.52)	*<* *0.001*	0.31 (0.19–0.52)	*<* *0.001*
LVI	No	1.0	–	–	–
	Yes	0.98 (0.72–1.34)	0.904	0.97 (0.71–1.33)	0.869
BVI	No	1.0	–	–	–
	Yes	1.19 (0.50–2.88)	0.693	1.13 (0.47–2.73)	0.787
pT stage	pT1	1.0	–	–	–
	pT2	1.76 (1.34–2.31)	*<* *0.001*	1.72 (1.31–2.26)	*<* *0.001*
	pT3	6.49 (3.87–10.89)	*<* *0.001*	6.31 (3.75–10.60)	*<* *0.001*
	pT4	2.18 (0.80–5.94)	0.129	1.96 (0.72–5.39)	0.190
pN stage	pN0	1.0	–	–	–
	pN1	0.78 (0.56–1.07)	0.123	0.79 (0.57–1.09)	0.149
	pN2	1.98 (1.22–3.20)	*0.006*	1.93 (1.19–3.14)	*0.008*
	pN3	3.25 (1.49–7.09)	*0.003*	3.22 (1.47–7.03)	*0.003*
	pN0	1.0	–	–	–
	pN1‑3	1.04 (0.79–1.36)	0.782	1.05 (0.80–1.38)	0.730

*BC* breast cancer, *BVI* blood vessel invasion, *CI* confidence interval, *DCIS* ductal carcinoma in situ, *ER* estrogen receptor, *HER2* human epidermal growth factor receptor 2, *IDC* invasive ductal carcinoma, *ILC* invasive lobular carcinoma, *LR* logistic regression, *LVI* lymphovascular invasion, *OR* odds ratio, *pN* pathological node stage, *PR* progesterone receptor, *pT* pathological tumor stage

^a^ adjusted for age at diagnosis

## Discussion

The aim of this study was to perform a comparison of the ALN status in ILC and IDC. In order to address this question, we retrospectively analyzed data of a national register, the Clinical Tumor Register (KTR) of the Austrian Association for Gynecological Oncology (AGO). A total of 2127 tumors were evaluated and compared in 2 groups (ILC *n* = 303, IDC *n* = 1824). In the multivariate analysis, pN2 and pN3 were observed significantly more frequently in ILC compared with IDC (OR: 1.93; 95% CI: 1.19–3.14; *p* = 0.008; OR: 3.22; 95% CI: 1.47–7.03; *p* = 0.003, respectively). Furthermore, multivariate analysis identified tumor grades 2 and 3, positive ER, and pT2 and pT3 as other factors associated with ILC. In contrast, a concomitant DCIS component, HER2-positivity, and moderate or high Ki67 were found less frequently in ILC compared to IDC. Taken together, our data show an increased risk of extensive axillary lymph node metastasis (pN2/3) in ILC.

In our study, mean age at diagnosis was significantly different (ILC: 65.0 years, IDC: 63.0 years, *p* = 0.01). Older age at diagnosis has been reported in several studies [6, 10, 11, 13, 17, 26, 31, 33]. Otherwise, age at diagnosis did not differ significantly between ILC and IDC [34–36]. Compared to patients with IDC, patients with ILC were less frequently premenopausal in our analysis; however, there are reports about no difference regarding the menopausal status in the literature [17, 33].

Patients with ILC presented more frequently with grade 2 tumors in comparison with IDC patients. This has been reported previously in several studies [13, 17, 29, 31]. According to our findings, more frequent ER-positivity in ILC was observed in different studies comparing these tumors with IDC [7, 9, 10, 16, 17, 29, 36]. We found more HER2-negative tumors with ILC, and several similar reports exist in the literature [1, 10, 12, 13, 37]. Our findings show a slow or moderate proliferation rate (Ki67) more frequently in lobular tumors. Other studies revealed that ILC was more often slowly proliferative [10, 12, 37]. In our analysis, there were no significant differences found according to bilateral BC, PR, lymphovascular and blood vessel invasion.

As in our study, ILC was associated with a larger tumor size in several trials [6–8, 10, 12, 13, 17, 29, 31, 38]; however, some studies found no difference in tumor size between ILC and IDC [16, 26, 35, 36].

In the descriptive analysis, we found a significantly different pN distribution between ILC and IDC. The multivariate analysis identified pN2 and pN3 as associated with ILC; however, published data on the ALN status in ILC compared with IDC are controversial [8, 10, 26–31]. Table 4 represents an overview of published data on the nodal status in association with the histological subtype.

**Table 4:** Published data on the ALN involvement in ILC compared with IDC

No difference in ALN involvement between ILC and IDC	Association between ILC and higher incidence of positive ALN involvement	Association between ILC and lower incidence of positive ALN involvement
Silverstein et al. (1994) [27]	Li et al. (2005) [38]	Sastre-Garau et al. (1996) [9]
Casolo et al. (1997) [39]	Wasif et al. (2010) [7]	Vandorpe et al. (2011) [31]
Mersin et al. (2003) [26]	Fernandez et al. (2011) [8]	Gao et al. (2020) [30]
Arpino et al. (2004) [10]	Chen et al. (2017) [13]	
Classe et al. (2004) [40]	Corona et al. (2020) [28]	
Pestalozzi et al. (2008) [17]	Danzinger et al. (2021) [29]	
Rakha et al. (2008) [11]		
Lee at al. (2010) [36]		
Jung et al. (2010) [12]		
Biglia et al. (2013) [16]		
Zengel et al. (2015) [33]		

*ALN* axillary lymph node, *IDC* invasive ductal carcinoma, *ILC* invasive lobular carcinoma

Most studies showed that the ALN involvement did not differ between these histopathological groups [10–12, 16, 17, 26, 27, 33, 36, 39, 40]. Arpino et al. [10] compared 4140 patients with ILC with 45,169 patients with IDC; there was no difference in the frequency of ALN involvement. Furthermore, nodal status did not differ between ILC and IDC in an analysis of 13,220 patients [17]; however, several studies found an association between ILC and a higher incidence of ALN positivity [7, 8, 13, 28, 29, 38]. Wasif et al. [7] demonstrated that ILC was more likely to be lymph node positive, when compared to IDC (36.8% versus 34.4%; *p* < 0.001). Evaluation of the Surveillance, Epidemiology and End Results Program (SEER) database showed a correlation between the ILC group and greater counts of positive lymph nodes [13]. In contrast, there are studies showing less frequent ALN involvement in ILC [9, 30, 31]. In a multivariate analysis, Vandorpe et al. [31] identified ILC as less likely to have ALN involvement (adjusted OR: 0.66; 95% CI: 0.53–0.82; *p* < 0.001). The authors retrospectively investigated 4292 tumors (ILC versus non-ILC). In contrast to our study, tumors with mixed ductal and lobular phenotypes were included. In conclusion, the lobular histology has been identified as an independent predictor of ALN involvement.

Limitations of this study are a moderate sample size and the retrospective design. Nevertheless, our results were similar to previously reported findings. In our analysis, we did not differentiate between the histological subtypes of ILC (e.g. classical, solid, alveolar); however, it is important to note that the patient collective of this investigation is quite homogeneous due to the inclusion and exclusion criteria: all patients underwent primary surgery, no patients with recurrent disease, or metastasis at the time of diagnosis.

In summary, this study was based on an Austrian cancer register. Our findings demonstrate an increased risk of extensive axillary lymph node metastasis (pN2/3) in ILC. Other factors associated with ILC were tumor grades 2 and 3, positive ER, and pT2 and pT3. In contrast, DCIS component, HER2+, and moderate or high Ki67 were found less frequently in ILC.

In the future, meta-analyses and pooling of data are required to make progress in research of ILC as a distinct histologic type of BC.

## Conclusion

The findings support an increased risk of extensive axillary lymph node metastasis (pN2/3) in ILC.

## Abbreviations

AGO: Association for Gynecological Oncology
ALN: Axillary lymph node
BC: Breast cancer
BVI: Blood vessel invasion
CI: Confidence interval
DCIS: Ductal carcinoma in situ
ER: Estrogen receptor
HER2: Human epidermal growth factor receptor 2
HR: Hormone receptor
IDC: Invasive ductal carcinoma
ILC: Invasive lobular carcinoma
KTR: Clinical Tumor Register, Klinisches TumorRegister
LVI: Lymphovascular invasion
OR: Odds ratio
PN: Pathological node stage
PR: Progesterone receptor
PT: Pathological tumor stage
SD: Standard deviation
SEER: Surveillance, Epidemiology, and End Results Program
